# Leaf venation networks of Bornean trees: images and hand‐traced segmentations

**DOI:** 10.1002/ecy.2844

**Published:** 2019-08-16

**Authors:** Benjamin Blonder, Sabine Both, Miguel Jodra, Noreen Majalap, David Burslem, Yit Arn Teh, Yadvinder Malhi

**Affiliations:** ^1^ Environmental Change Institute School of Geography and the Environment University of Oxford South Parks Road Oxford OX1 3QY UK; ^2^ School of Biological Sciences University of Aberdeen Zoology Building, Tillydrone Avenue Aberdeen AB24 2TZ UK; ^3^ Forest Research Centre Sabah Forestry Department Peti Surat 1407 90715 Sandakan, Sabah Malaysia; ^4^ Present address: School of Life Sciences Arizona State University Tempe Arizona USA; ^5^ Present address: School of Environmental and Rural Science University of New England Armidale New South Wales Australia

**Keywords:** botany, cleared leaf, ecology, image analysis, image segmentation, leaf venation, machine learning, plant ecophysiology, tropical forest, tropical forest, vein network, venation network

## Abstract

The data set contains images of leaf venation networks obtained from tree species in Malaysian Borneo. The data set contains 726 leaves from 295 species comprising 50 families, sampled from eight forest plots in Sabah. Image extents are approximately 1 × 1 cm, or 50 megapixels. All images contain a region of interest in which all veins have been hand traced. The complete data set includes over 30 billion pixels, of which more than 600 million have been validated by hand tracing. These images are suitable for morphological characterization of these species, as well as for training of machine‐learning algorithms that segment biological networks from images. Data are made available under the Open Data Commons Attribution License. You are free to copy, distribute, and use the database; to produce works from the database; and to modify, transform, and build upon the database. You must attribute any public use of the database, or works produced from the database, in the manner specified in the license. For any use or redistribution of the database, or works produced from it, you must make clear to others the license of the database and keep intact any notices on the original database.

The complete data sets corresponding to abstracts published in the Data Papers section in the journal are published electronically as Supporting Information in the online version of this article at http://onlinelibrary.wiley.com/doi/10.1002/ecy.2844/suppinfo.

## Data Availability

Because of file size, data are permanently available at the Oxford Research Archive at https://ora.ox.ac.uk/objects/uuid:de65fc07-4b8f-4277-a6c4-82836afbdeb3.

