# Supplementary material for: Leaf venation networks of Bornean trees: images and hand‐traced segmentations
Source: Ecology. 2019 Aug 16;100(11):e02844. doi: 10.1002/ecy.2844 (PMC6899953; doi:10.1002/ecy.2844)
Supplement: Supplementary file 1 [file ECY-100-na-s001.pdf]

Title.
Leaf venation networks of Bornean trees: images and hand-traced segmentations

Authors
Benjamin Blonder (bblonder@gmail.com)
Address: Environmental Change Institute, School of Geography and the Environment, University of Oxford, Oxford, United Kingdom
Present address: School of Life Sciences, Arizona State University, Tempe, Arizona, United States of America

Sabine Both
Address: School of Biological Sciences, University of Aberdeen, Aberdeen, United Kingdom
Present address: School of Environmental and Rural Science, University of New England, Armidale, New South Wales, Australia

Miguel Jodra
Address: Environmental Change Institute, School of Geography and the Environment, University of Oxford, Oxford, United Kingdom

Noreen Majalap
Address: Forest Research Centre, Sabah Forestry Department, Sandakan, Sabah, Malaysia

David Burslem
Address: School of Biological Sciences, University of Aberdeen, Aberdeen, United Kingdom

Yit Arn Teh
Address: School of Biological Sciences, University of Aberdeen, Aberdeen, United Kingdom

Yadvinder Malhi
Address: Environmental Change Institute, School of Geography and the Environment, University of Oxford, Oxford, United Kingdom

Metadata

1. **Class I. Data set descriptors**
   a. **Data set identity:** Leaf venation networks of Bornean trees: images and hand-traced segmentations
   b. **Data set identification code:** https://ora.ox.ac.uk/objects/uuid:de65fc07-4b8f-4277-a6c4-82836afbdeb3
   c. **Data set description**
      i. **Originator:** University of Oxford Ecosystems Laboratory
      ii. **Period of study:** Field work was conducted in late 2015 and was concluded by early 2016. Lab analyses were carried out during 2016. All image analyses were carried out during 2016 and 2017.
      iii. **Abstract:** The data set contains images of leaf venation networks obtained from tree species in Malaysian Borneo. The data set contains 726 leaves from 295 species comprising 50 families, sampled from 8 forest plots in Sabah. Image extents are approximately 1 × 1 cm, or 50 megapixels. All images contain a region of interest in which all veins have been hand-traced. The complete data set includes over 30 billion pixels, of which more than 600 million have been validated by hand-tracing. These images are suitable for morphological characterization of these species, as well as for training of machine-learning algorithms that segment biological networks from images. Data are made available under the Open Data Commons Attribution License. You are free to copy, distribute and use the database, to produce works from the database, and to modify, transform and build upon the database. You must attribute any public use of the database, or works produced from the database, in the manner specified in the license. For any use or redistribution of the database, or works produced from it, you must make clear to others the license of the database and keep intact any notices on the original database.
   d. **Key words.** leaf venation, tropical forest, image segmentation, machine learning, cleared leaf, vein network, venation network, botany, tropical forest, ecology, image analysis, plant ecophysiology
2. **Class II. Research origin descriptors**
   a. Overall project description
      i. **Objectives:** Leaves have diverse venation networks with architecture that varies widely, from a single vascular strand (e.g. pines) to purely branching structures (e.g. gingko) to open net patterns (e.g. many ferns) to mostly parallel structures in monocots (e.g. corn) to highly reticulate patterns in many angiosperms (e.g. lemon) (Roth-Nebelsick et al. 2001, Sack and Scoffoni 2013). Architecture is closely linked to functions of transport, mechanical support, and resistance/resilience to damage (Brodribb et al. 2016, Ronellenfitsch and Katifori 2017, Blonder et al. 2018). Variation in architecture across scales may have emerged in response to multiple selective factors across ecological contexts and phylogenetic history. A growing body of literature (e.g. (Brodribb et al. 2016, Rishmawi et al. 2017, Ronellenfitsch and Katifori 2017, Blonder et al. 2018)) is now investigating the architecture of these networks.

However, empirical understanding of the causes and consequences of variation in network architecture has been limited by the availability of quantitative data for analyses. It is time-intensive to obtain images of leaf venation networks, to segment these images into binary representations of veins and not-veins, and to extract useful statistics from these segmentations. Several algorithms have been proposed to address the segmentation and extraction challenges (e.g. (Parsons-Wingerter and Vickerman 2011, Dhondt et al. 2012, Katifori and Magnasco 2012, Price and Weitz 2014, Bühler et al. 2015, Ronellenfitsch et al. 2015, Lasser and Katifori 2017)), but it has been unclear how to compare among algorithms. This problem is relevant both to the immediate needs of the plant ecophysiology and systematics research communities, but also more broadly to the computer science and biomimicry research communities focused on understanding the architecture of spatial transportation networks in general. In this case, leaf venation networks present an important test case for algorithms aimed at generating realistic networks, for algorithms aimed at segmenting networks from images, and for algorithms aimed at extracting useful descriptors of networks from segmentations.

To address all of these issues, it is important to make available image data sets with broad morphological/phylogenetic coverage, and to make available validated ('ground truth') segmentations of these image data sets. Such information could better characterize the empirical variation observed in leaf venation networks in nature, as well as providing a standardized test case for different competing algorithms. From such validated data it is possible to calculate confusion matrices (e.g. number of true/false negatives/positives) and thus metrics of algorithm performance. A growing number of segmentation and extraction algorithms are now being developed that use concepts from the artificial intelligence and machine learning fields (Fu and Chi 2006, Ronneberger et al. 2015, Xu et al. in preparation, Brodrick et al. in review). These types of algorithms must be trained on a large amount of validated data to learn their task, and often have much better performance than achieved through other approaches. In many other areas (e.g. object-segmentation/classification) standardized validated data sets already exist (e.g. The Open Images data set, which contains 9.2 million images containing 15 million bounding-boxes for 600 object types (Kuznetsova et al. 2018)) and are commonly used to test novel algorithms. No such resource presently exists for spatial transportation networks.

Motivated by these issues, we present a data set comprising high-resolution images and segmentations of leaf venation networks of 726 leaves from 295 species. The complete data set includes more than 30

billion pixels and includes more than 600 million validated pixels that have been hand-classified via image tracing.

b. Specific subproject description

i. **Site description:** Samples were obtained from eight permanent forest plots that are part of the Global Ecosystems Monitoring network (http://gem.tropicalforests.ox.ac.uk) and Smithsonian CTFS (Center for Tropical Forest Science) network (https://forestgeo.si.edu). Funding for this activity was provided by the UK Natural Environment Research Council (NERC) as part of the research activities of the Biodiversity And Land-use Impacts on tropical ecosystem function (BALI) research consortium (http://bali.nerc-hmtf.info). All project data are archived with the SAFE Project (https://www.safeproject.net/) at https://zenodo.org/communities/safe.

The plots were selected to span a logging intensity gradient and are characterized by a mixed dipterocarp lowland forest in various stages of regeneration. Each plot is 1 hectare in size, with all stems ≥ 10 cm diameter at breast height tagged. Sites are fully described in (Both et al. 2018) and (Riutta et al. 2018).

ii. **Experimental or sampling design:** Leaves were sampled from the species comprising the top 70% of the total plot basal area. Because this sampling strategy disproportionately selects for large and abundant species, we also sampled trees with a stratified random design to capture rare and smaller species. Three 20 × 20 m subplots were selected within every permanent plot and all trees within a subplot sampled. Sunlit and/or shaded leaves from each stem were sampled from each stem.

Species were identified by MinSheng Kooh, Bernadu Bala Ola, and Alexander Karolus and are linked to voucher specimens from the same trees that are stored at the Danum Valley field herbarium (Sabah, Malaysia). All taxonomic identifications in the master collection list were made by experts based on voucher specimens (Both et al. 2018). Specimens are stored permanently at the Danum Valley Study centre herbarium. Data for tree locations and sizes can be found in the corresponding information of the permanent plots, archived in the Forest Plots database (www.forestplots.net). Plot names are shown in **Figure 2**.

Each sample has a unique 'branch code' that can be truncated to a unique 'tree code' (see section IV.b for details), e.g. branch 'DAS1-T010542-BSH' corresponds to tree 010542 within the DAS1 site. As such, the taxonomic hypotheses underlying this study can be traced and updated in the future.

A wide range of functional traits, covering physical, chemical and physiological properties measured at tree- or branch-level as part of this field campaign are also available separately using the same branch codes.

These traits are described in (Both et al. 2018) and are publicly archived at https://zenodo.org/communities/safe and also available at http://dx.doi.org/10.5281/zenodo.3247631 (individual-level trait values) and http://dx.doi.org/10.5281/zenodo.3247602 (community weighted mean trait values).

For venation networks, each leaf was cleaned using a wet rag and then pressed flat and dried at 60°C for at least one week. 1 cm^2 sections were cut from each leaf, selecting a segment at the midpoint of the leaf (from base to apex) and midway between the margin and midvein on the left side (viewed abaxially). Leaf sections were then chemically cleared following standard protocols for dried leaf tissue (Pérez-Harguindeguy et al. 2013). This approach may potentially yield biased images due to leaf shrinkage after the initial drying process. However, leaf shrinkage is fully reversible via rehydration (Blonder et al. 2012), which was the first step of the standard leaf clearing protocol used here (Pérez-Harguindeguy et al. 2013).

Briefly, leaf samples were immersed in warm 5% aqueous sodium hydroxide until transparent (3-7 days), rinsed in water, and then bleached in 2.5% aqueous sodium hypochlorite for 5 min. After another water rinse, samples were passed through an ethanol dehydration series to 100% ethanol, then lignin stained in 0.1% safranin in ethanol. After a 100% ethanol rinse, samples were passed through a dilution series to 100% toluene and then mounted on a glass slide in a toluene-based mounting medium (Richard-Allen Scientific).

After allowing slides to dry for 3 days, each leaf was imaged using a compound microscope (Olympus, BX43) with 2x apochromat objective and a color camera (3840 x 2748 pixel resolution; Olympus, SC100). 9-16 overlapping image fields were stitched together for each sample to obtain a complete image of the sampled area using GIMP software (GNU).

Images were pre-processed in MATLAB (MathWorks) by retaining the green channel of each image which has the greatest contrast after safranin staining, and applying contrast-limited adaptive histogram equalization, with a 400×400 pixel window and a contrast limit of 0.01.

A validated region-of-interest (ROI) of approximately 700×700 pixels was manually traced for each image, using a digitizing tablet (Wacom, Cintiq 22HD) and GIMP software. All the veins within this region were traced, with the traced vein widths corresponding to the apparent widths in images. Another image mask was also drawn to include only useful pixels, i.e. excluding background and damaged pixels. Last, any substantive veins with width greater than approximately 0.5 mm were also manually delineated through the entire image, as these were not routinely included

in tracings.

The final data set contains 726 leaves from 295 species comprising 50 families. All images have a resolution of 595 pixels per millimeter.

Figure 1: Number of samples **(A)** or the number of species **(B)** obtained across taxonomic families.

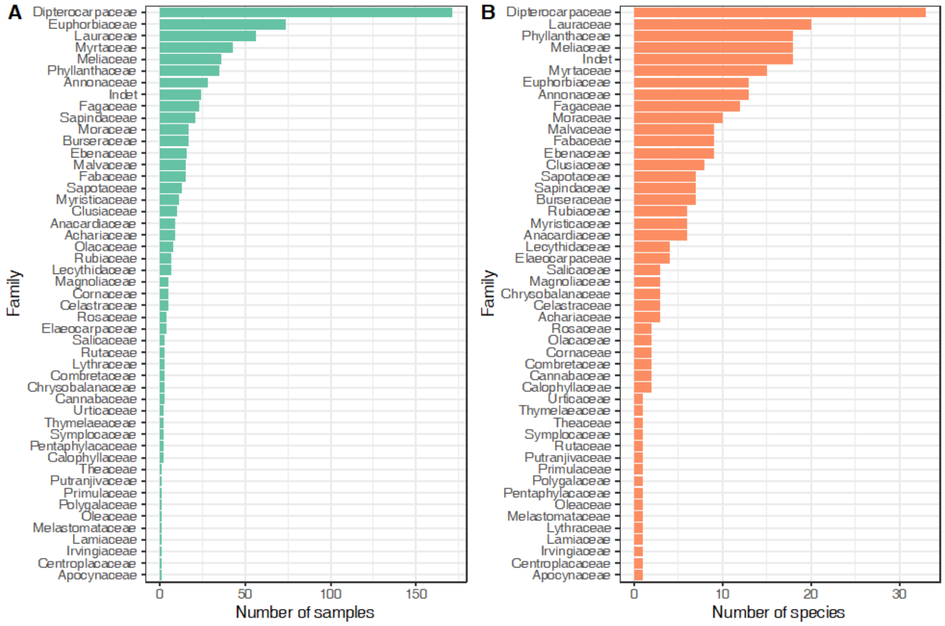

Figure 2: Number of samples obtained across each of the eight sites.

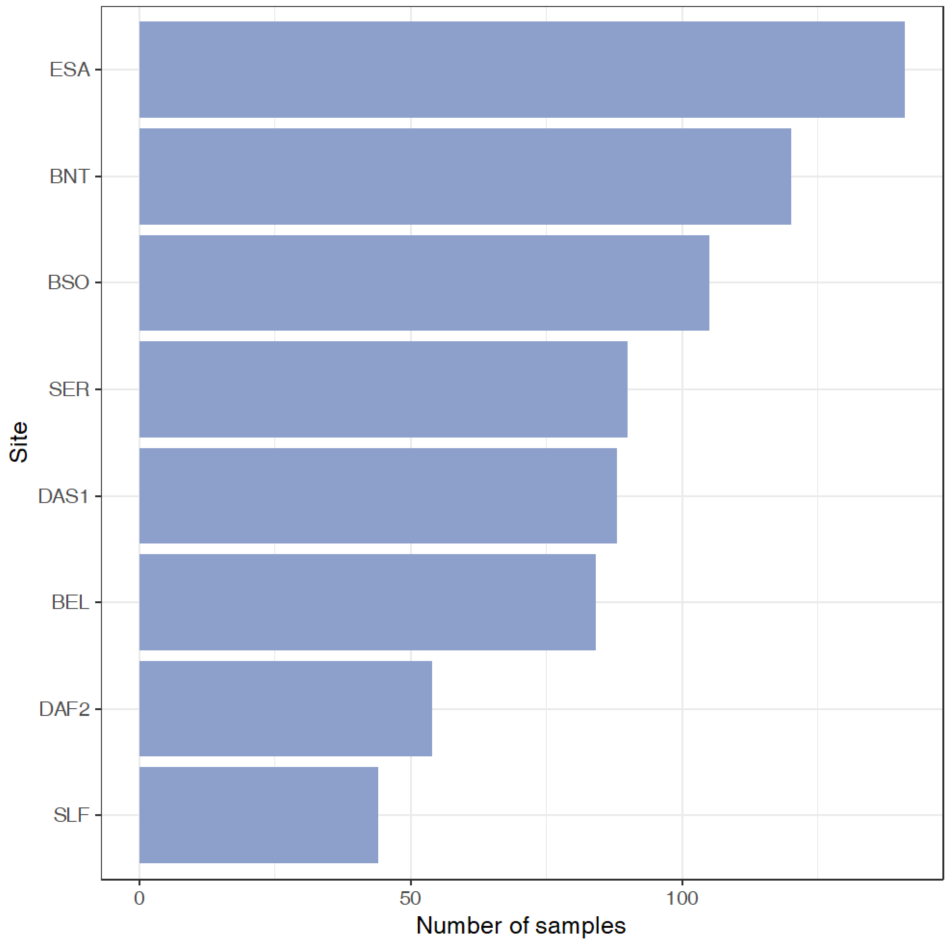

The data set has coverage of a large number of plant families, though the depth of sampling across families is variable (**Figure 1**). The Dipterocarpaceae, Lauraceae, Phyllanthaceae, and Euphorbiaceae are especially well-represented in terms of number of samples and species, reflecting their overall abundance (**Figure 2**). Sampling coverage per species is given in **Table 1**.

Table 1. Number of samples per species across all sites.

Species	Count
Actinodaphne borneensis	1
Adinandra dumosa	2
Aglaia crassinervia	1
Aglaia leptantha	1
Aglaia macrocarpa	2
Aglaia odoratissima	2
Aglaia oligophylla	3
Aglaia silvestris	3
Aglaia tomentosa	1
Alangium javanicum	4
Alstonia angustiloba	1
Antiaris toxicaria	1
Antidesma stipulare	1

Aphanamixis polystachya	1
Aporosa confusa	1
Aporosa falcifera	1
Aporosa illustris	1
Aquilaria beccariana	2
Archidendron clypearia	1
Ardisia macrophylla	1
Artocarpus anisophyllus	4
Artocarpus glaucus	1
Artocarpus integer	1
Artocarpus odoratissimus	1
Artocarpus tamaran	1
Atuna racemosa	1
Baccaurea lanceolata	1
Baccaurea latifolia	1
Baccaurea macrocarpa	2
Baccaurea tetrandra	5
Barringtonia lanceolata	2
Barringtonia macrostachya	2
Barringtonia sarcostachys	1
Beilschmiedia cuadrae	1
Beilschmiedia micrantha	2
Bhesa paniculata	1
Callicarpa pentandra	1
Calophyllum soulattri	1
Calophyllum woodii	1
Canarium decumanum	4
Canarium denticulatum	4
Canarium odontophyllum	1
Canarium pilosum	1
Caryodaphnopsis tonkinensis	2
Castanopsis hypophoenicea	3
Chionanthus macrocarpus	1
Chisocheton ceramicus	6
Chisocheton macranthus	1
Chisocheton patens	3
Chisocheton sarawakanus	3
Cleistanthus hirsutulus	3
Cleistanthus hylandii	3
Cleistanthus indet	1
Cleistanthus myrianthus	3
Cleistanthus paxii	1
Cleistanthus pubens	1
Clidemia hirta	3
Cratoxylum cochinchinense	2
Cromolaena odorata	1
Crudia reticulata	1
Crudia tenuipes	1
Cryptocarya nigra	1
Cryptocarya nitens	1
Cyathocalyx deltoideus	1
Cynometra mirabilis	1
Dacryodes rostrata	1
Dacryodes rugosa	3
Dehaasia caesia	2
Dehaasia incrassata	1
Dendrocnide elliptica	2
Dialium indum	3
Dialium kunstleri	3
Dicranopteris pubigera	1
Dimocarpus longan	2
Dinochloa trichogona	1

Diospyros andamanica	1
Diospyros curranii	1
Diospyros demona	3
Diospyros dictyoneura	1
Diospyros macrophylla	5
Diospyros muricata	1
Diospyros pilosanthera	1
Diospyros toposia	1
Diospyros tuberculata	2
Dipterocarpus caudiferus	4
Dryobalanops lanceolata	8
Drypetes longifolia	1
Duabanga moluccana	3
Durio grandiflorus	2
Durio graveolens	2
Dysoxylum cauliflorum	2
Dysoxylum cyrtobotryum	2
Dysoxylum densiflorum	1
Elaeocarpus floribundus	1
Elaeocarpus pedunculatus	1
Elaeocarpus stipularis	1
Etlingera spec	1
Eusideroxylon zwageri	22
Ficus hispida	1
Ficus septica	4
Ficus uncinata	1
Ficus variegata	2
Flacourtia rukam	1
Fordia brachybotrys	1
Fordia splendidissima	1
Garcinia benthamiana	1
Garcinia forbesii	1
Garcinia nervosa	2
Garcinia parvifolia	1
Gironniera nervosa	1
Glochidion borneensis	1
Glochidion elmeri	1
Glochidion lutescens	6
Glochidion rubrum	2
Gluta aptera	1
Gluta wallichii	3
Heritiera elata	1
Homalium foetidum	1
Hopea plagata	2
Hopea sangal	1
Horsfieldia crassifolia	2
Hydnocarpus anomalus	4
Hydnocarpus polypetalus	4
Hydnocarpus woodii	1
Imperata cylindrica	1
Indet indet	2
Irvingia malayana	1
Jacquemontia tomentella	1
Kayea oblongifolia	1
Knema latifolia	1
Knema laurina	2
Knema oblongata	2
Knema pulchra	1
Lansium domesticum	1
Licania splendens	1
Lindera lucida	3
Lithocarpus blumeanus	1

Lithocarpus conocarpus	1
Lithocarpus echinifer	2
Lithocarpus gracilis	3
Lithocarpus leptogyne	2
Lithocarpus orocola	1
Lithocarpus sundaicus	2
Lithocarpus urceolaris	2
Litsea accedens	4
Litsea angulata	5
Litsea caulocarpa	1
Litsea garciae	2
Litsea grandis	2
Litsea mappacea	1
Litsea rubiginosa	1
Lophopetalum beccarianum	2
Lophopetalum glabrum	1
Lophopetalum javanicum	2
Ludekia borneensis	2
Maasia sumatrana	8
Macaranga conifera	1
Macaranga gigantea	7
Macaranga hypoleuca	4
Macaranga pearsonii	36
Macaranga winkleri	1
Madhuca dubardii	2
Madhuca korthalsii	2
Maesa macrothyrsa	1
Magnolia borneensis	1
Magnolia liliifera	2
Magnolia tsiampacca	2
Mallotus leucodermis	7
Mallotus mollissimus	1
Mallotus penangensis	1
Mallotus wrayi	5
Mangifera odorata	1
Mastixia trichotoma	1
Melanochyla bullata	1
Melanochyla tomentosa	1
Melastoma malabathricum	1
Melicope confusa	3
Memecylon oleaefolium	1
Merremia borneensis	1
Mesua borneensis	1
Mesua macrantha	1
Microcos crassifolia	1
Mikania micrantha	3
Miliusa macropoda	1
Monoon hookerianum	1
Myristica smythiesii	3
Nauclea officinalis	1
Neo-uvaria acuminatissima	1
Neolamarckia cadamba	1
Neonauclea gigantea	1
Neoscortechinia kingii	3
Neoscortechinia philippinensis	1
Nephelium cuspidatum	1
Nephelium ramboutan-ake	6
Nephrolepsis biserrata	1
Nothaphoebe umbelliflora	2
Ochanostachys amentacea	7
Orophea myriantha	2
Palaquium dasyphyllum	4

Palaquium obovatum	1
Palaquium sericeum	1
Paranephelium macrophyllum	2
Paranephelium xestophyllum	6
Parashorea malaanonan	7
Parashorea smythiesii	1
Parashorea tomentella	12
Parinari oblongifolia	1
Parishia insignis	2
Paspalum virgatum	1
Payena acuminata	2
Payena microphylla	1
Pentace laxiflora	4
Phaeanthus splendens	1
Phoebe grandis	1
Phrynium pubinerve	1
Planchonia brevistipitata	2
Pleiocarpus polyneura	1
Polyalthia obliqua	4
Pometia pinnata	3
Prunus beccarii	2
Prunus javanica	2
Pseuduvaria borneensis	1
Psydrax dicoccos	1
Pterygota alata	2
Ptychopyxis arborea	5
Pyrenaria tawauensis	1
Quercus argentata	4
Quercus lowii	1
Quercus merrillii	1
Reinwardtiodendron humile	2
Ryparosa acuminata	1
Sageraea elliptica	1
Santiria laevigata	3
Scaphium macropodum	1
Scorodocarpus borneensis	1
Shorea agami	1
Shorea almon	5
Shorea angustifolia	9
Shorea argentifolia	2
Shorea beccariana	5
Shorea dasyphylla	4
Shorea faguetiana	5
Shorea fallax	10
Shorea gibbosa	9
Shorea guiso	2
Shorea johorensis	9
Shorea laevis	2
Shorea leprosula	6
Shorea leptoderma	2
Shorea macrophylla	3
Shorea macroptera	6
Shorea ovalis	4
Shorea ovata	4
Shorea parvifolia	9
Shorea parvistipulata	1
Shorea pauciflora	14
Shorea superba	1
Shorea symingtonii	2
Shorea xanthophylla	3
Sindora irpicina	3
Sloanea javanica	1

Spathiostemon javensis	2
Spatholobus macropterus	1
Stelechocarpus cauliflorus	1
Sterculia rubiginosa	1
Sterculia stipulata	1
Symplocos fasciculata	2
Syzygium caudatilimbum	1
Syzygium chloranthum	1
Syzygium elopurae	2
Syzygium grande	8
Syzygium griffithii	2
Syzygium kunstleri	15
Syzygium lineatum	1
Syzygium myrtifolium	2
Syzygium napiforme	1
Syzygium pancheri	3
Syzygium perpuncticulatum	1
Syzygium racemosum	1
Syzygium rheophyticum	1
Syzygium tenuicaudatum	3
Terminalia citrina	2
Terminalia foetidissima	1
Trema orientalis	2
Trigonobalanus verticillata	1
Tristaniopsis whiteana	1
Tristiropsis acutangula	1
Uncaria cordata	2
Vatica dulitensis	14
Vatica odorata	4
Walsura pinnata	1
Xanthophyllum flavescens	1
Xylopia ferruginea	1
Xylopia stenopetala	5

Each sample is represented by a large amount of image data. The median ground-truthed region of interest comprises 698,688 pixels (interquartile range 445,121, 1,138,850) that are classified as either vein or not-vein. The median useful image extent comprises 45,417,957 pixels (interquartile range 40,300,352, 50,328,031). The complete data set contains 686,881,432 total ground-truth pixels, as well as 32,815,701,653 total useful pixels.

Figure 3: Histogram of **(A)** the number of validated pixels or **(B)** the number of total useful pixels (i.e. within the overall image mask) across all samples. Vertical red lines indicate distribution medians.

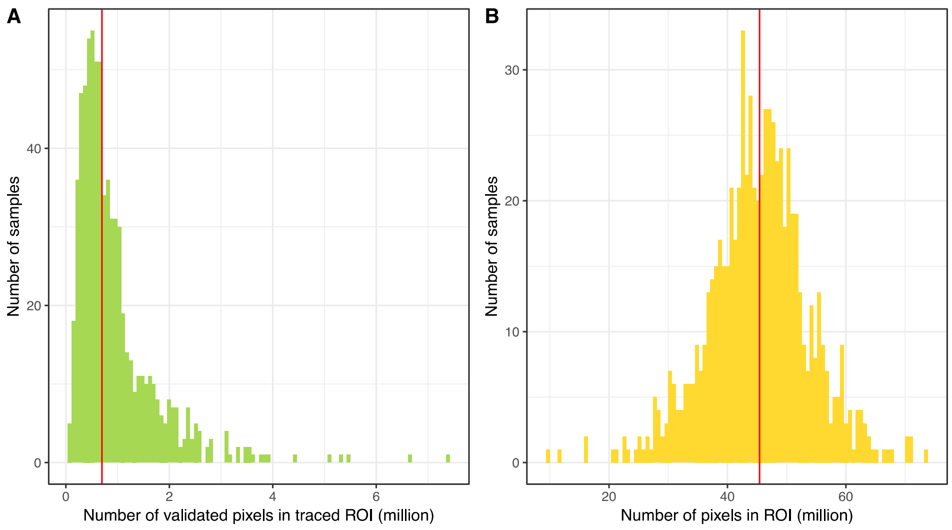

3. **Class III. Data set status and accessibility**
   a. **Status**
      i. **Latest update:** 14 December 2018 (version 1.0)
   b. Accessibility
      i. **Storage location and medium:** Data are permanently available at the Oxford Research Archive at https://ora.ox.ac.uk/objects/uuid:de65fc07-4b8f-4277-a6c4-82836afbdeb3. Physical specimens are stored at the Ecosystems Laboratory, School of Geography and the Environment, University of Oxford, South Parks Road, Oxford, OX1 3QY, United Kingdom.
      ii. **Contact person(s):** Benjamin Blonder; bblonder@gmail.com
      iii. **Copyright restrictions:** Data are made available under the Open Data Commons Attribution License (http://opendatacommons.org/licenses/by/). You are free to copy, distribute and use the database, to produce works from the database, and to modify, transform and build upon the database. You must attribute any public use of the database, or works produced from the database, in the manner specified in the license. For any use or redistribution of the database, or works produced from it, you must make clear to others the license of the database and keep intact any notices on the original database.
4. **Class IV. Data structural descriptors**
   a. **Data set file**
      i. **Identity:** Blonder_B_2018_Leaf_venation_networks_Data.zip
      ii. **Size:** 24846.9 megabytes
      iii. **Format and storage mode:** The ZIP archive expands to a directory containing 3,216 files, comprising 3,215 portable network graphic (PNG) images and 1 comma separated value (CSV) metadata file.
   b. **Variable information**
   Each sample has a unique CODE, of the format <*a*>-T<*b*>-B<S|SH> where *a* represents the name of a permanent plot, *b* the unique tag identifier of a stem within the plot, and S if the sampled branch was sunlit or SH if shaded. This

CODE can be used to cross reference the sample to other associated data available in trait and plot databases (Both et al. 2018). See Section II.b.ii for references to trait data sets.

The site codes used in the metadata file indicate field codes and must be translated to the codes used in these other databases:

Site code (this database)	Site code (ForestPlots)	Site name
DAS1	DAN-04	Danum GEM Carbon Plot 1
DAF2	DAN-05	Danum GEM Carbon Plot 2
BEL	MLA-01	Maliau SAFE GEM Carbon Plot: Belian
SER	MLA-02	Maliau SAFE GEM Carbon Plot: Seraya
BSO	SAF-01	SAFE GEM Carbon Plot B South
BNT	SAF-02	SAFE GEM Carbon Plot B North
ESA	SAF-03	SAFE GEM Carbon Plot E
SLF	SAF-04	SAFE GEM Carbon Plot LF

In this data set, each sample is represented by 4-5 image files which are named,
<CODE>_img.png: the raw grayscale image
<CODE>_roi.png: a binary mask indicating the hand-traced region of interest
<CODE>_seg.png: a binary mask indicating the hand-traced presence/absence of veins within the region of interest
<CODE>_slc.png: a binary mask indicating the hand-traced presence/absence of leaf tissue within the image (of high image quality, i.e. excluding tears, bubbles, dust)
<CODE>_big.png: a binary mask indicating the hand-traced presence/absence of larger veins within the region of interest.
The first 4 files are always present for each sample; the last only when it was determined that it was necessary to trace larger veins. Examples of the type of imagery in the data set are shown in **Figure 4**.

A metadata file linking each sample to its taxonomy is also present (Metadata.csv). The file includes the following columns:
CODE: the unique sample code described above; variable-length character format
Family: the taxonomic family of the sample; variable-length character format
Species: the Latin binomial species name of the sample; variable-length character format

Figure 4. Example samples from the data set for **(A)** code DAS1-T010542-BSH, species *Terminalia citrina* (Combretaceae), **(B)** code DAS1-T050189-BS, species *Lophopetalum javanicum* (Celastraceae), **(C)** code BNT-T245-BS, species *Artocarpus odoratissimus* (Moraceae), **(D)** code SLF-T52-BS, species *Actinodaphne borneensis* (Lauraceae). Panels from left to right show the full image, the useful pixel mask, the ground-truth mask, a zoom of the image cropped to the ground-truth mask, and the ground-truth classification. Images have differing extents but are shown at the same size; the red scale bar in each image is 1 mm wide.

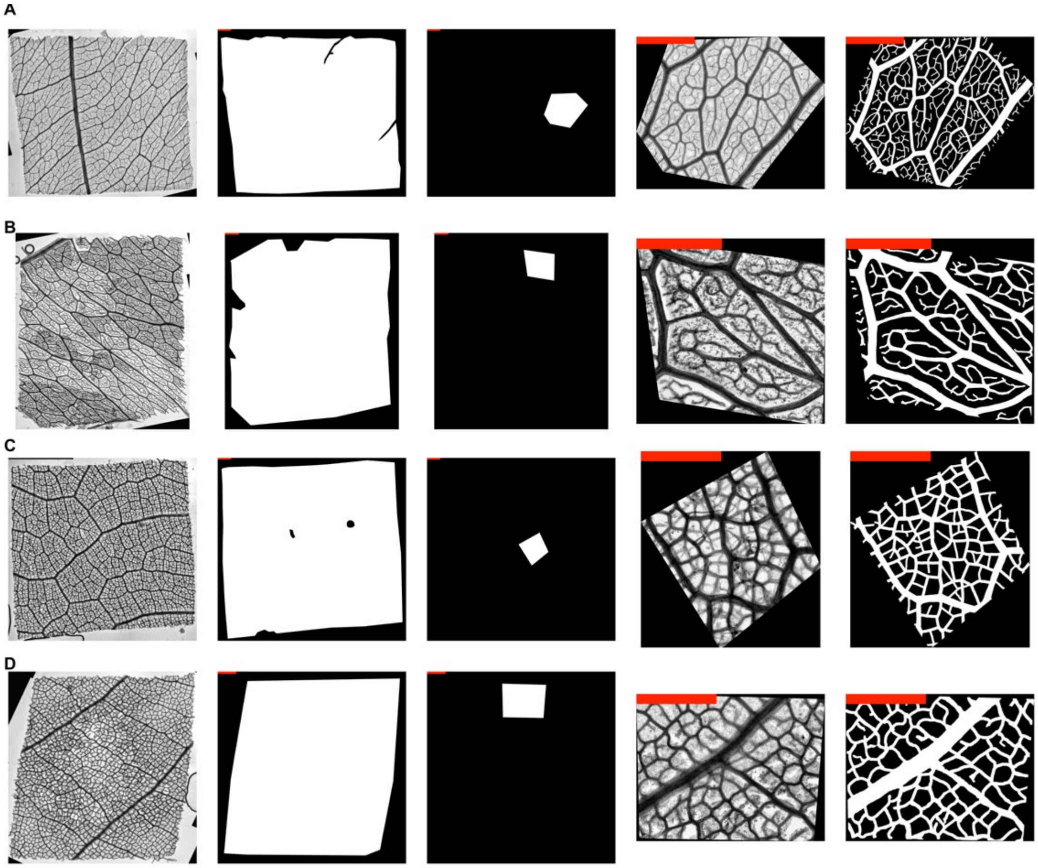

c. **Missing value codes:** Samples where the family was not able to be determined have the Family field coded as 'Indet'.'
d. **Known issues:** In the metadata, species coded as *Pleiocarpus polyneura* should be *Pleiocarpidia polyneura*.

5. **Class V. Supplemental descriptors**
   a. **Data acquisition**
      i. **Data entry verification procedures** - Codes for leaf samples were checked against master collection lists for mislabeling problems immediately after samples were processed in the field, before histological preparation, and after collection of image data. Any errors were resolved by comparison against the master list using contextual information and/or by assuming the master code with minimum Hamming distance relative to the observed code was correct.
   b. **Quality assurance/quality control procedures -** Ground-truth tracings were made by a team of people who received a daylong training in venation network morphology from the project's lead (B. Blonder). All tracings were then supervised by the project technician (M. Jodra) in consultation with the project lead. Every tracing was assessed by at least two people, with any disagreements discussed verbally and then hand-corrected by the team.
   c. **Archiving**
      i. **Archival procedures:** Tracings were originally processed in GIMP (GNU) image editing software and then exported to platform-independent

formats. Files were archived with the University of Oxford's Oxford Research Archive in December 2018.

d. **Publications and results:** More information on the underlying field campaign that generated these data is described in (Both et al. 2018).

e. **History of data set usage**

    i. **Data set update history:** 14 December 2018 - version 1.0 - initial release.

Acknowledgments
We thank Unding Jami, Matheus Henrique Nuñes, Rudi Saul Cruz Chino, and Milenka Ximena Montoya for their assistance with fieldwork, and Rob Ewers, Laura Kruitbos, Reuben Nilus, Glen Reynolds, and Charles Vairappan for facilitating research in Malaysia. This work was supported by the UK Natural Environment Research Council (NERC) (#NE/M019160/1, to BB) and the US National Science Foundation (#DEB-1840209, to BB). This publication is a contribution from the NERC Human-modified Tropical Forest Programme (#NE/M017508/1, to YAT) and Biodiversity And Land-use Impacts on Tropical Ecosystem Function (BALI) consortium (#NE/K016253/1, to YM and #NE/K016253/1, to YAT). The SAFE Project was funded by the Sime Darby Foundation and the UK NERC. The study areas are part of the Global Ecosystems Monitoring Network (GEM) via an ERC Advanced Investigator Award to YM (#321131). We acknowledge support from the Stability of Altered Forest Ecosystems (SAFE) Project, the Sabah Biodiversity Council (SaBC), the Institute for Tropical Biology and Conservation (ITBC) at the University of Malaysia, Sabah (UMS), the Sabah Forest Research Centre (FRC) at Sepilok, the Sabah Forestry Department, the South East Asia Rainforest Research Program, Yayasan Sabah (Maliau Basin Conservation Area), and the Maliau Basin and Danum Valley Management Committees.

Literature cited

Blonder, B., V. Buzzard, I. Simova, L. Sloat, B. Boyle, R. Lipson, B. Aguilar-Beaucage, A. Andrade, B. Barber, C. Barnes, D. Bushey, P. Cartagena, M. Chaney, K. Contreras, M. Cox, M. Cueto, C. Curtis, M. Fisher, L. Furst, J. Gallegos, R. Hall, A. Hauschild, A. Jerez, N. Jones, A. Klucas, A. Kono, M. Lamb, J. D. R. Matthai, C. McIntyre, J. McKenna, N. Mosier, M. Navabi, A. Ochoa, L. Pace, R. Plassmann, R. Richter, B. Russakoff, H. S. Aubyn, R. Stagg, M. Sterner, E. Stewart, T. T. Thompson, J. Thornton, P. J. Trujillo, T. J. Volpe, and B. J. Enquist. 2012. The leaf-area shrinkage effect can bias paleoclimate and ecology research. American Journal of Botany **99**:1756-1763.

Blonder, B., N. Salinas, L. Patrick Bentley, A. Shenkin, P. Chambi Porroa, Y. Valdez Tejeira, T. Boza Espinoza, G. R. Goldsmith, L. Enrico, R. Martin, G. P. Asner, S. Díaz, B. Enquist, and Y. Malhi. 2018. Structural and defensive roles of angiosperm leaf venation network reticulation across an Andes-Amazon elevation gradient. J Ecol.

Both, S., T. Riutta, C. E. T. Paine, D. M. O. Elias, R. S. Cruz, A. Jain, D. Johnson, U. H. Kritzler, M. Kuntz, N. Majalap-Lee, N. Mielke, M. X. Montoya Pillco, N. J. Ostle, Y. Arn Teh, Y. Malhi, and D. F. R. P. Burslem. 2018. Logging and soil nutrients independently explain plant trait expression in tropical forests. New Phytologist **221**:1853-1865.

Brodribb, T. J., D. Bienaimé, and P. Marmottant. 2016. Revealing catastrophic failure of leaf networks under stress. Proceedings of the National Academies of Sciences **113**:4865–4869.

Brodrick, P. G., A. B. Davies, and G. P. Asner. in review. Uncovering Ecological Patterns with Convolutional Neural Networks. Trends in Ecology and Evolution **in press**.

Bühler, J., L. Rishmawi, D. Pflugfelder, G. Huber, H. Scharr, M. Hülskamp, M. Koornneef, U. Schurr, and S. Jahnke. 2015. phenoVein-A tool for leaf vein segmentation and analysis. Plant physiology:pp. 00974.02015.

Dhondt, S., D. Van Haerenborgh, C. Van Cauwenbergh, R. M. Merks, W. Philips, G. T. Beemster, and D. Inzé. 2012. Quantitative analysis of venation patterns of Arabidopsis leaves by supervised image analysis. The Plant Journal **69**:553-563.

Fu, H., and Z. Chi. 2006. Combined thresholding and neural network approach for vein pattern extraction from leaf images. IEE Proceedings-Vision, Image and Signal Processing **153**:881-892.

Katifori, E., and M. O. Magnasco. 2012. Quantifying loopy network architectures. PLoS ONE **7**:e37994.

Kuznetsova, A., H. Rom, N. Alldrin, J. Uijlings, I. Krasin, J. Pont-Tuset, S. Kamali, S. Popov, M. Malloci, and T. Duerig. 2018. The open images dataset v4: Unified image classification, object detection, and visual relationship detection at scale. arXiv preprint arXiv:1811.00982.

Lasser, J., and E. Katifori. 2017. NET: a new framework for the vectorization and examination of network data. Source code for biology and medicine **12**:4.

Parsons-Wingerter, P., and M. B. Vickerman. 2011. Informative mapping by VESGEN analysis of venation branching pattern in plant leaves such as Arabidopsis thaliana. Gravitational and Space Research **25**.

Pérez-Harguindeguy, N., S. Díaz, E. Garnier, S. Lavorel, H. Poorter, P. Jaureguiberry, M. Bret-Harte, W. Cornwell, J. Craine, and D. Gurvich. 2013. New handbook for standardised

measurement of plant functional traits worldwide. Australian Journal of botany **61**:167-234.

Price, C. A., and J. S. Weitz. 2014. Costs and benefits of reticulate leaf venation. BMC Plant Biology **14**:234.

Rishmawi, L., J. Bühler, B. Jaegle, M. Hülskamp, and M. Koornneef. 2017. Quantitative trait loci controlling leaf venation in Arabidopsis. Plant, cell & environment **40**:1429-1441.

Riutta, T., Y. Malhi, L. K. Kho, T. R. Marthews, W. Huaraca Huasco, M. Khoo, S. Tan, E. Turner, G. Reynolds, S. Both, D. F. R. P. Burslem, Y. A. Teh, C. S. Vairappan, N. Majalap, and R. M. Ewers. 2018. Logging disturbance shifts net primary productivity and its allocation in Bornean tropical forests. Global Change Biology **24**:2913-2928.

Ronellenfitsch, H., and E. Katifori. 2017. The Phenotypes of Fluctuating Flow: Development of Distribution Networks in Biology and the Trade-off between Efficiency, Cost, and Resilience. arXiv:1707.03074.

Ronellenfitsch, H., J. Lasser, D. C. Daly, and E. Katifori. 2015. Topological Phenotypes Constitute a New Dimension in the Phenotypic Space of Leaf Venation Networks. PLoS Computational Biology **11**:e1004680.

Ronneberger, O., P. Fischer, and T. Brox. 2015. U-net: Convolutional networks for biomedical image segmentation. Pages 234-241 *in* International Conference on Medical image computing and computer-assisted intervention. Springer.

Roth-Nebelsick, A., D. Uhl, V. Mosbrugger, and H. Kerp. 2001. Evolution and function of leaf venation architecture: a review. Annals of Botany **87**:553-566.

Sack, L., and C. Scoffoni. 2013. Leaf venation: structure, function, development, evolution, ecology and applications in the past, present and future. New Phytologist **198**:983-1000.

Xu, H., M. Fricker, and B. Blonder. in preparation. Convolutional neural network image segmentation for biological transportation networks.
